# Pediatric Precursor B-Cell Lymphoblastic Malignancies: From Extramedullary to Medullary Involvement

**DOI:** 10.3390/cancers14163895

**Published:** 2022-08-12

**Authors:** Emma Kroeze, Laura Arias Padilla, Max Bakker, Judith M. Boer, Melanie M. Hagleitner, Birgit Burkhardt, Takeshi Mori, Andishe Attarbaschi, Jaime Verdú-Amorós, Marta Pillon, Liliya Anderzhanova, Edita Kabíčková, Alan K. S. Chiang, Rejin Kebudi, Karin Mellgren, Jelena Lazic, Janez Jazbec, Jules P. P. Meijerink, Auke Beishuizen, Jan L. C. Loeffen

**Affiliations:** 1 Princess Máxima Center for Pediatric Oncology, 3584 CS Utrecht, The Netherlands; 2 NHL-BFM Study Center and Pediatric Hematology and Oncology, University Hospital Muenster, 48149 Muenster, Germany; 3 Department of Hematology and Oncology, Hyogo Prefectural Kobe Children’s Hospital, Kobe 650-0047, Japan; 4 Department of Pediatric Hematology and Oncology, St. Anna Children’s Hospital, Medical University of Vienna, 1090 Vienna, Austria; 5 Pediatric Oncology Department, Hospital Clínico Universitario de Valencia, 46010 Valencia, Spain; 6 Clinic of Pediatric Hemato-Oncology, Department of Women’s and Children’s Health, University of Padova, 46010 Padova, Italy; 7 Dmitry Rogachev National Medical Research Center of Pediatric Hematology, Oncology and Immunology, 117198 Moscow, Russia; 8 Department of Pediatric Hematology and Oncology, Charles University, 2nd Medical School and University Hospital Motol, 150 06 Prague, Czech Republic; 9 Department of Pediatrics and Adolescent Medicine, Li Ka Shing Faculty of Medicine, The University of Hong Kong, Queen Mary Hospital, Pokfulam, Hong Kong; 10 Division of Pediatric Hematology-Oncology, Istanbul University, Oncology Institute, 34452 İstanbul, Turkey; 11 Department of Pediatric Oncology, Sahlgrenska University Hospital, University of Gothenburg, 413 45 Gothenburg, Sweden; 12 Department for Hematology and Oncology, University Children’s Hospital, University of Belgrade, 11000 Belgrade, Serbia; 13 Division of Pediatrics, Hematology and Oncology, University Medical Center Ljubljana, SI-1000 Ljubljana, Slovenia; 14 Erasmus Medical Center, Sophia Children’s Hospital, 3015 CN Rotterdam, The Netherlands

**Keywords:** B-cell lymphoblastic lymphoma, BCP-LBL, B-cell acute lymphoblastic leukemia, BCP-ALL, non-Hodgkin lymphoma, NHL, disease spectrum

## Abstract

**Simple Summary:**

B-cell lymphoblastic lymphoma (BCP-LBL) and B-cell acute lymphoblastic leukemia (BCP-ALL) are both malignancies of immature B-cells. However, BCP-ALL has been extensively studied and treatment protocols have changed over the last decades, whereas BCP-LBL is quite rare, and treatment has stayed roughly the same. In this retrospective study, we compare the clinical characteristics of a cohort of BCP-LBL patients to a cohort BCP-ALL patients. With the comparison of this unique large cohort of immature B-cell malignancies, we aim to contribute to elucidating whether BCP-LBL and BCP-ALL represent two diseases, or different representations of the same disease. Increasing the understanding of BCP-LBL in comparison to BCP-ALL is crucial for improving treatment and prognosis for BCP-LBL.

**Abstract:**

B-cell lymphoblastic lymphoma (BCP-LBL) and B-cell acute lymphoblastic leukemia (BCP-ALL) are the malignant counterparts of immature B-cells. BCP-ALL is the most common hematological malignancy in childhood, while BCP-LBL accounts for only 1% of all hematological malignancies in children. Therefore, BCP-ALL has been well studied and treatment protocols have changed over the last decades, whereas treatment for BCP-LBL has stayed roughly the same. Clinical characteristics of 364 pediatric patients with precursor B-cell malignancies were studied, consisting of BCP-LBL (*n* = 210) and BCP-ALL (*n* = 154) patients. Our results indicate that based on the clinical presentation of disease, B-cell malignancies probably represent a spectrum ranging from complete isolated medullary disease to apparent complete extramedullary disease. Hepatosplenomegaly and peripheral blood involvement are the most important discriminators, as both seen in 80% and 95% of the BCP-ALL patients and in 2% of the BCP-LBL patients, respectively. In addition, we show that the overall survival rates in this cohort differ significantly between BCP-LBL and BCP-ALL patients aged 1–18 years (*p* = 0.0080), and that the outcome for infants (0–1 years) with BCP-LBL is significantly decreased compared to BCP-LBL patients of all other pediatric ages (*p* < 0.0001).

## 1. Introduction

Precursor B-cell lymphoblastic malignancies comprise B-cell acute lymphoblastic leukemia (BCP-ALL) and B-cell lymphoblastic lymphoma (BCP-LBL). Whereas BCP-ALL is the most common hematological malignancy in children (40%), BCP-LBL is currently seen as a rare hematological disease accounting for only 1% of all childhood hematological malignancies [1,2]. As defined by the World Health Organization, a lymphoblastic malignancy with ≥25% blasts in bone marrow (BM) at diagnosis is considered BCP-ALL, while extramedullary disease and cases with a medullary blast count of less than 25% are defined as BCP-LBL [3]. Precursor B-cell malignancies probably represent a spectrum, ranging from solely medullary involvement with no signs of extramedullary disease to apparently complete extramedullary disease with minimal or no BM involvement. These two main subgroups are derived from the transformation of precursor B-cells and are indistinguishable based on morphology/immunohistochemistry and immunophenotype. Contrary to BCP-ALL, genetic mutations have not been extensively studied in children with BCP-LBL. However, candidate gene approach studies showed that several cytogenetic abnormalities seen in BCP-ALL occur in BCP-LBL as well, e.g., *ETV6-RUNX1* fusions and a hyperdiploid karyotype [4,5,6].

The similarities between BCP-LBL and BCP-ALL support the hypothesis that both disease entities manifest from the same cell of origin. Given their presumed common origin and biological similarities, BCP-LBL treatment is typically derived from ALL-based protocols. In contrast to BCP-ALL regimens, in which treatment intensity is driven by the presence of somatic molecular genetic aberrations as well as by the assessment of minimal residual disease (MRD), BCP-LBL treatment remained roughly the same in the last two decades due to lack of established predictive molecular biomarkers and sensitive response criteria [7]. Survival rates of children with BCP-LBL and BCP-ALL are favorable, but the outcome of relapsed BCP-LBL patients remains dismal [8,9]. This illustrates the need for improved risk stratification for BCP-LBL.

Based on the hypothesis that BCP-LBL and BCP-ALL originate from the same cell and that these malignancies can represent a spectrum instead of two different disease entities, we compared the clinical characteristics of 210 BCP-LBL patients to 154 BCP-ALL patients. The comparison of this unique large cohort of immature B-cell malignancies can contribute to elucidate these enigmas.

## 2. Patients and Methods

### 2.1. B-Cell Lymphoblastic Lymphoma

Data from 210 BCP-LBL patients were obtained by means of a standard case report form. Patients diagnosed between 1999 and 2019 (1999–2009: *n* = 60; 2009–2019: *n* = 118, unknown: *n* = 5) were retrieved from 14 EICNHL and/or i-BFM Study Group members (Germany, *n* = 72; Japan, *n* = 31; the Netherlands, *n* = 23; Spain, *n* = 20; Austria, *n* = 18; Italy, *n* = 12; Russian Federation, *n* = 10; Czech Republic, *n* = 7; Hongkong, *n* = 7; Turkey, *n* = 5; Switzerland, *n* = 4; Sweden, *n* = 3; Serbia, *n* = 1; Slovenia, *n* = 1). Date of diagnosis was unknown for five patients. Stage of disease was recorded as Murphy stage [10]. Most patients were treated according to the NHL-BFM based protocols, including the international EICNHL-EURO-LB02 protocol (Supplementary Figure S1). Other protocols (ALB-NHL03, regional protocols and ALL-treatment protocols) are highly comparable to EURO-LB02, for which patients with Murphy/St Jude stage I and II disease are treated with lower intensity than patients with stage III and IV disease [10].

### 2.2. B-Cell Acute Lymphoblastic Leukemia

Data from 154 BCP-ALL patients for comparison were consecutively included from two hospitals in the Netherlands. All BCP-ALL patients aged ≥1 years old were treated according to the Dutch Childhood Oncology Group ALL10 protocol [11] in the Erasmus Medical Center in Rotterdam and University Medical Center Utrecht, between 2004 and 2012. In this protocol, a thoracic X-ray and an abdominal ultrasound were mandatory in all patients. Characteristics of patients included in the ALL10 protocol are comparable to patients included in other protocols, including the ALL-BFM95 and the UKALL2003 protocol, showing the suitability of this cohort for this study [12,13]. Infants with ALL aged <1 years were treated according to a specific infant protocol and not included for comparison in this study.

The requested data for both cohorts included demographics, clinical characteristics at diagnosis and cytogenetic data. Collected variables are described in Supplementary Table S1. Data obtained from these patients included age, sex, examination for lymphadenopathy, radiology results from a thoracic X-ray and abdominal ultrasound (mandatory for all patients in the ALL10 protocol), blood values at diagnosis, the percentage of blasts in the bone marrow (BM) and peripheral blood (PB) and central nervous system (CNS) involvement, as indicated by CNS status. Lymphadenopathy was based on an enlargement of at least 10 mm or when confirmed malignant in a pathology report. For both BCP-LBL and BCP-ALL, CNS status was defined as follows: CNS1 as ≤5 white blood cells (WBC)/µL in the cerebral spinal fluid (CSF) without leukemic cells, CNS2 as ≤5 WBC/µL CSF with identifiable leukemic cells and CNS involvement as CNS3 as >5 WBC/µL CSF with identifiable leukemic cells or intracerebral/meningeal masses, cranial nerve palsy or retinal involvement. Traumatic lumbar puncture (TLP) is defined as the presence of red blood and leukemic cells, both in a specific ratio.

### 2.3. Statistical Analysis

Event-free survival (EFS) and overall survival (OS) were calculated from date of initial diagnosis to date of first event (i.e., relapse, refractory disease, secondary malignancy or death of any cause) and/or date of last known follow-up. The survival curves were computed using the Kaplan–Meier method and the differences between the curves were analyzed for statistical significance using the log-rank test (Graphpad prism v8, San Diego, CA, USA). Risk factors were determined by performing a Cox proportional hazards test and differences between dichotomous variables were analyzed with Fisher’s Exact test (Graphpad prism v8, San Diego, CA, USA). Statistical significance between hematological values at diagnosis was analyzed by using the Mann–Whitney test (Graphpad prism v8, San Diego, CA, USA).

## 3. Results

### 3.1. Patient Characteristics at Diagnosis

**Figure 1:**
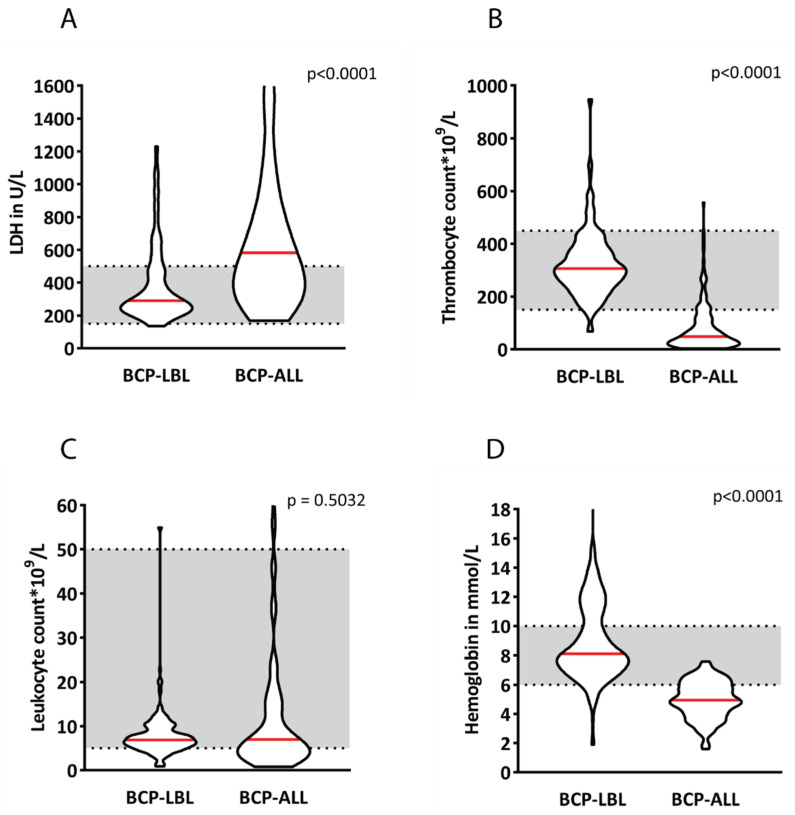
Violin plots of blood values at diagnosis for 210 B-cell lymphoblastic lymphoma (BCP-LBL) and 154 B-cell acute lymphoblastic leukemia (BCP-ALL) patients showing median values (red line) of LDH (**A**), thrombocyte counts (**B**), leukocyte counts (**C**) and hemoglobin (**D**). Thickness of the violins represent the number of patients.

The patients’ characteristics are summarized in Table 1. The median age at diagnosis was 6.1 years (range 0–18 years) for the BCP-LBL patients and 4.6 years (range 1.2–17.5 years) for the BCP-ALL patients. The median age of BCP-LBL patients was significantly higher (*p* < 0.0001) even though infants were included, whereas this was not the case for the BCP-ALL patients. The male/female (M/F) ratio was comparable for BCP-LBL and BCP-ALL with 1.3:1 (57% males) and 1.1:1 (52% males), respectively. Most of the BCP-LBL patients were diagnosed with advanced disease stage (Murphy [10]) (*n* = 204): stage I (9%), stage II (19%), stage III (36%) and stage IV (33%). Median hematological values at diagnosis (i.e., hemoglobin, lactate dehydrogenase levels, leukocyte counts and thrombocyte counts) except for leukocyte counts, differed significantly between BCP-LBL and BCP-ALL (*p* < 0.0001). For BCP-LBL, all values were within the physiological range with values of 8.1 mmol/L, 290 U/L, 6.9 × 10^9^/L, and 306 × 10^9^/L, respectively. These values were, with the exception of leukocyte counts, out of the physiological range for BCP-ALL, with 5 mmol/L, 711 U/L, 7.0 × 10^9^/L, and 48 × 10^9^/L, respectively (Figure 1). 

### 3.2. Localizations of B-Cell Malignancies

**Figure 2:**
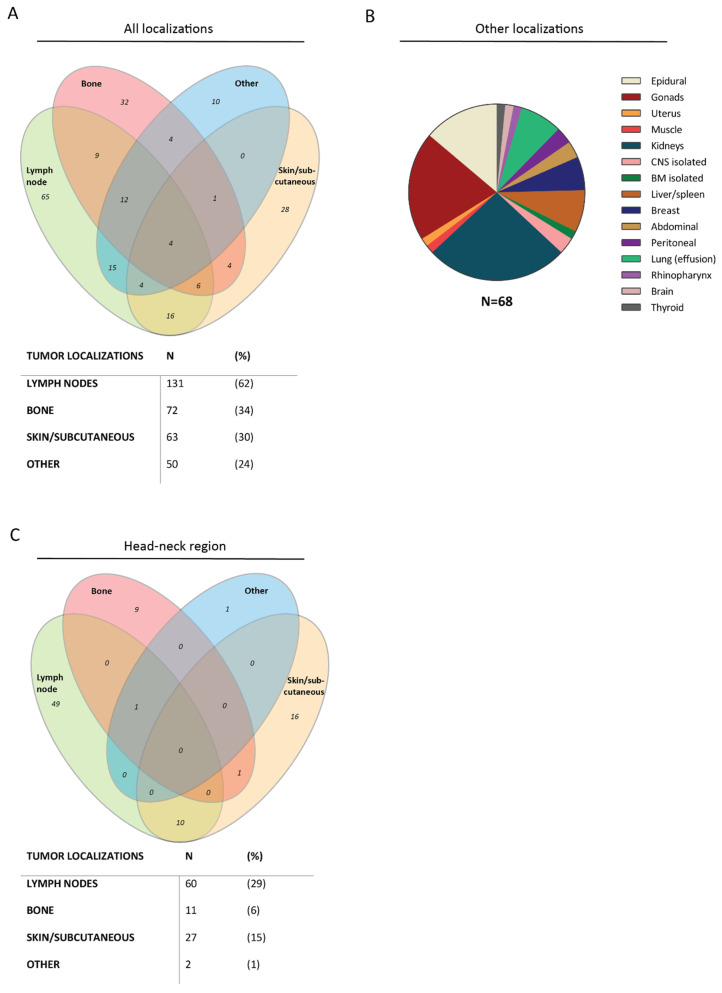
BCP-LBL lesions occur mainly in the lymph nodes, bone and skin/subcutaneous regions (**A**). Localizations other than the three main categories (**B**). Localizations in head–neck region. possibly with a preference for head–neck region (**C**).

BCP-ALL is characterized by extensive bone marrow (BM) involvement (range 28–98% blasts) in all patients and by peripheral blood (PB) involvement (range 0–96% blasts) in 95% of the patients. However, only 26% of the BCP-LBL presented with BM involvement (cytomorphological 5–25% blasts), and 3% of the patients presented with blasts in the PB (range 1–31% blasts) (Table 2). For BCP-LBL, extramedullary localizations were more common. The localizations were divided in three main groups: lymph nodes, bone lesions and skin/subcutaneous lesions (Figure 2A). The most common site of disease in our BCP-LBL cohort was the lymph node, affecting 131 patients (62%), followed by bone lesions in 72 patients (34%). The bone lesions occurred in both multifocal and unifocal, and patients with BM involvement (26%) were not overrepresented in the bone lesion group, or any other group. The third most common site of disease was subcutaneous masses/skin lesions, in 63 patients (30%). In addition to these main categories, 50 patients (24%) had lesions that could not be designated to any one of these groups (Figure 2B). All patients could be represented in different groups, due to multiple affected tissues.

Strikingly, many of the aforementioned lesions were located in the head–neck region (Figure 2C) (100/315 lesions): 29% of the patients had lymph node involvement in the head–neck region, 6% of the patients had a facial bone lesion and 15% of the patients had a facial skin lesion/subcutaneous mass. Two patients had a lesion in or on the head–neck region that could not be designated to the three main tissue categories (i.e., rhinopharynx and frontal lobe). Involvement of the CNS (CNS2: ≤5 WBC/µL CSF with identifiable leukemic cells or CNS3: >5 WBC/µL CSF with identifiable leukemic cells) was observed in 20% of the BCP-LBL cohort, based on morphology.

Of the BCP-ALL patients, 32% (49/154) had an enlarged (suspected pathological) lymph node, assessed by physical examination and/or imaging. Seven BCP-ALL patients (5%) presented with extranodal masses; six subcutaneous masses in the head–neck region and one testicular mass. Hepato- and/or splenomegaly was seen in 80% (123/154) of the BCP-ALL patients. In contrast, hepato- and/or splenomegaly was reported in only 2% of the BCP-LBL patients (Table 2).

Radiological involvement of the kidneys was seen in 11% of the BCP-ALL and 7% of BCP-LBL patients. Half of the BCP-ALL patients in our cohort showed a CNS2 status (based on cytomorphology) but merely one of the patients had CNS3, concordant with CNS2 and CNS3 occurring in about 42% and 1% of all patients in the complete Dutch DCOG ALL10 study, respectively [11].

### 3.3. Cytogenetic Aberrations

Cytogenetic data in BCP-LBL are scarce and cytogenetic analyses in this cohort were not routinely performed at diagnosis. We obtained cytogenetic data including karyotyping and fluorescent in situ hybridization (FISH) from 15 patients. Aberrations that were identified included *KMT2A*-rearrangements (*n* = 4), an *IgH-MYC* translocation (*n* = 1), *ETV6-RUNX1* fusions (*n* = 2), intrachromosomal amplification of chromosome 21 (iAMP21) (*n* = 1) and a *CCND3-ETV6* fusion (*n* = 1). In addition, karyotyping showed a gain of chromosome 21 in one patient and a (subclonal) hyperdiploid karyotype in six patients. Cytogenetic investigation was performed in all patients in the ALL-10 cohort. Especially *ETV6-RUNX1* fusions and hyperdiploidy were often found, in 33 (21%) and 58 (38%) BCP-ALL patients, respectively.

### 3.4. Differences in Overall Survival between BCP-LBL and BCP-ALL

**Figure 3:**
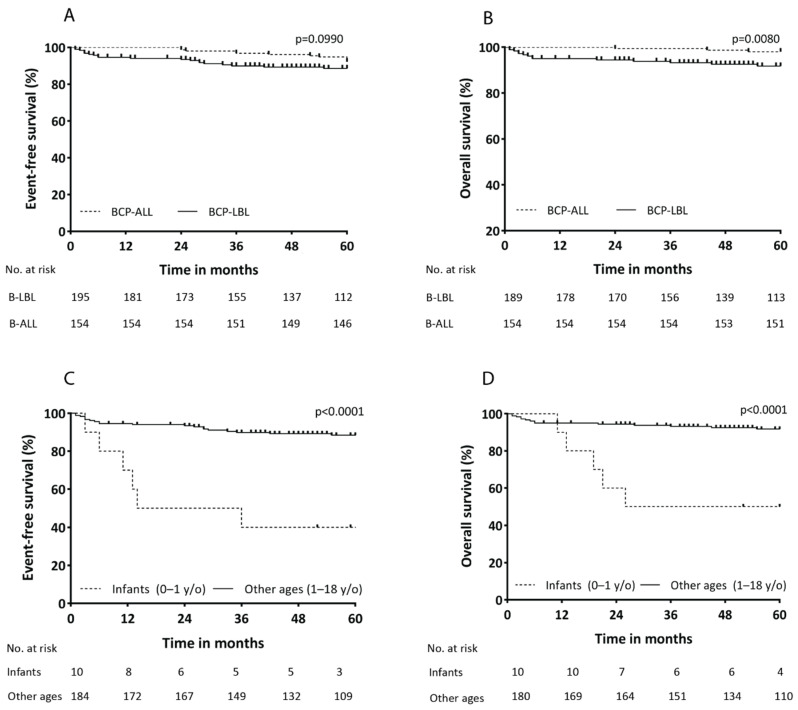
Event-free and overall survival for BCP-LBL patients compared to BCP-ALL patients (aged 1–18 years) (**A**,**B**) and infants with BCP-LBL compared to the other ages (**C**,**D**).

Overall, the outcome for precursor B-cell malignancies is favorable. The 5-year event-free survival (EFS) and overall survival (OS) rates for BCP-LBL were 86% ± 3% and 90% ± 2%, respectively (Supplementary Figure S2A,B). For BCP-ALL, 5-year event-free survival (EFS) and overall survival (OS) rates were 92% ± 2% and 98% ± 1%, respectively (Supplementary Figure S2C,D). When comparing all patients aged 1–18 years, there was a significant difference between BCP-LBL and BCP-ALL with OS rates of 92% ± 2% and 98% ± 1%, respectively (*p* = 0.0080) (Figure 3A,B). Different LBL treatment protocols did not lead to differences in survival (Supplementary Figure S2E,F). The survival curves for the different disease stages (stage I/II/III compared to stage IV) showed a trend toward inferior OS for stage IV disease (Supplementary Figure S2G,H), as expected.

Of the patients aged 1–18 years old, 13 BCP-LBL patients had a relapse (6%), with a median time to relapse of 24 months (range, 3–142 months) after diagnosis. Only one patient had a very late relapse after 5 years. Relapse was reported in 11 (7%) BCP-ALL patients, with a median time to relapse of 52 months (range 24–84 months). Late relapse (more than 36 months after initial diagnosis) was seen in 9/11 BCP-ALL patients. Of the relapsed BCP-ALL patients, 64% patients achieved a second complete remission after chemotherapeutic treatment, while of the relapsed BCP-LBL patients 69% did. The causes of death for the BCP-LBL patients were progressive disease (*n* = 3) or relapse (*n* = 5), treatment-related toxicity (*n* = 6) and secondary hematological disease (*n* = 2). For BCP-ALL, the causes of death were progressive disease (*n* = 1) and relapse (*n* = 2).

### 3.5. Inferior Outcome for Infants with BCP-LBL

In addition to the 13 relapsed BCP-LBL patients aged 1–18 years, 6/10 infants (<1 years old) diagnosed with BCP-LBL relapsed as well, comprising 32% of all relapse cases. The median time to relapse was 12 months (range 3–36 months). Among the relapsed infants, the mortality rate was 100%. The median time to death was 20 months (range 11–63 months). This high number of infants that had a relapse is represented by a significant inferior EFS and OS for infants; comparing infants (<1 years old at diagnosis) (*n* = 10) and children with BCP-LBL of all other ages (≥ 1 years old) (*n* = 184), the survival rates were significantly poorer, with a 5-year EFS of 40% ± 15% for infants and 88% ± 2% for other ages (HR 6.9, 95% CI [1.0–47.8] (*p* < 0.0001)) and a 5-year OS of 44% ± 16% and 92% ± 2% (HR 9.0, 95%CI [1.1–73.4] (*p* < 0.0001)) (Figure 3C,D).

## 4. Discussion

**Figure 4:**
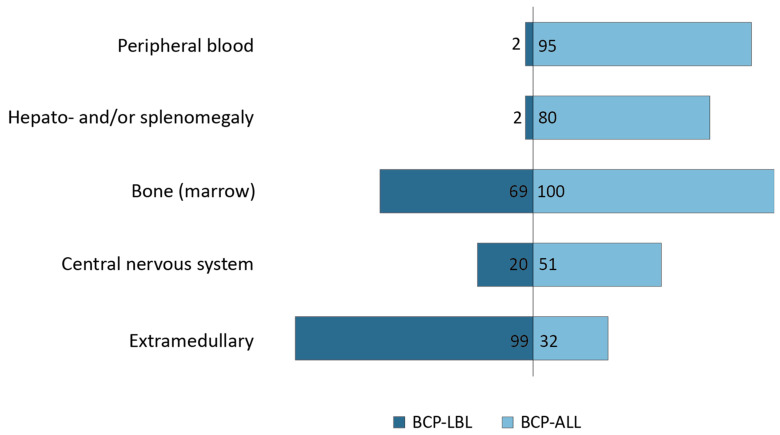
Model of disease localizations showing that there is an overlap in disease presentation between BCP-LBL and BCP-ALL for which BCP-LBL typically presents extramedullary and BCP-ALL in hematopoietic organs.

B-cell lymphoblastic lymphoma (BCP-LBL) and B-cell lymphoblastic leukemia (BCP-ALL) arise from the malignant expansion of precursor B-cells. Even though close overlap between the two disease entities is expected, the exact clinical and molecular biological relationships between BCP-LBL and BCP-ALL remain to be elucidated. BCP-ALL is characterized by disseminated disease and involvement of the hematopoietic organs (BM, PB, and liver/spleen), but extramedullary lesions are seen in about one-third of the BCP-ALL patients as well. Conversely, BCP-LBL is typically localized extramedullary, but about one-third of the patients also present with disseminated disease. When combining imaging data with BM aspirations, we show that 69% of the BCP-LBL patients present with bone (or BM) involvement. Therefore, we propose a model suggesting that B-cell malignancies express a clinical continuum that may range from complete isolated medullary disease to apparent complete extramedullary disease, with currently blasts in PB and hepatosplenomegaly as most important discriminators (Figure 4).

Despite the fact that BCP-LBL and BCP-ALL are closely related, overall survival rates for children 1–18 years old were significantly lower for the BCP-LBL patients (92% ± 2%) compared to the BCP-ALL patients (98% ± 1%) in this cohort (*p* = 0.0080). However, the BCP-ALL patients in this study have slightly higher OS and EFS rates than reported in the complete ALL10 study [11]. Even though this outcome is very similar, stratification in BCP-LBL is not based on MRD and molecular aberrations, in contrast to BCP-ALL. Therefore, it is impossible to draw any conclusions from this comparison [11]. Contemporary childhood BCP-ALL-treatment protocols are stratified based on MRD and somatic genetic risk factors, whereas BCP-LBL treatment protocols are mainly stage-based since high-risk cytogenetics for BCP-LBL are still unknown. These BCP-LBL treatment protocols are derived from BCP-ALL-treatment protocols but have stayed roughly the same over the last decades. This is due to a lack of molecular genetic risk factors in BCP-LBL, as well as a lack of stratification based on treatment response [7].

It is known that infants with (*KMT2A*-rearranged) BCP-ALL have a decreased outcome compared to BCP-ALL patients of all other ages, and therefore infants with BCP-ALL are treated according to infant treatment protocols in most major European study groups and large pediatric oncology centers outside Europe [14,15]. We show that the infants with BCP-LBL had significantly decreased EFS rates and OS rates compared to the patients of all other ages with BCP-LBL as well. They were treated according to the same protocol as BCP-LBL patients of all other ages. The 6-year EFS and OS were 49% and 58%, respectively, for BCP-ALL infants treated in the Interfant-06 protocol [16], which is higher than the 5-year estimates for BCP-LBL infants in the current study (i.e. 33% EFS and 44% OS). Cytogenetically, infant BCP-ALL is often characterized by *KMT2A* rearrangements, combined with frequent co-expression of myeloid markers [17]. It is uncertain whether *KMT2A* rearrangements characterize infant BCP-LBL as well. In our BCP-LBL cohort, FISH analysis for *KMT2A* rearrangements was performed in 3/9 of the infant cases and this genetic aberration was not identified in any of the patients. Further exploration is needed to investigate whether infants with BCP-LBL would benefit from other treatment regimens as well.

In order to fully elucidate whether BCP-LBL and BCP-ALL represent a disease spectrum rather than two different diseases, more extensive cytogenetic analyses are indispensable. Based on the (scarce) cytogenetic data in this cohort, there is an overlap in the chromosomal abnormalities observed in the BCP-LBL compared to known aberrations in BCP-ALL. However, this has been determined by FISH analyses for known BCP-ALL aberrations. All aberrations found in this cohort were previously described in childhood BCP-ALL, where *KMT2A* rearrangements are seen in ~6% of the patients, a hyperdiploid karyotype and *ETV6-RUNX1* fusions in ~20–25% of the patients and iAMP21 in ~2% of the patients [18]. However, *IgH-MYC* translocations are rare and seen in <1% of the BCP-ALL patients and interestingly, the *CCND3-ETV6* fusion is extremely rare and described in only one ALL patient [19]. This rare translocation and fusion in BCP-LBL were observed in this small cohort of only 15 samples; the actual frequency should be confirmed in a larger cohort. Elucidating the genetic drivers of BCP-LBL may identify low- and high-risk factors for improved risk stratification in BCP-LBL. Additionally, MRD-risk stratification and the role of an FDG-PET/CT scan at day 33 should be explored for improved stratification in BCP-LBL [20]. Exploration of differences in genetic aberrations between BCP-LBL and BCP-ALL may explain the course of disease and therefore clinical presentation of BCP-LBL and BCP-ALL.

## 5. Conclusions

In contrast to BCP-ALL, there are currently no evidence-based BCP-LBL treatment protocols stratified on molecular genetic risk factors and/or treatment response available. The stratification of BCP-ALL patients led to superior survival curves, while OS rates for BCP-LBL stayed roughly the same in the last decades. Changing perspectives on whether BCP-ALL and BCP-LBL present a spectrum rather than two different diseases, and the search for MRD targets as well as molecular risk factors in BCP-LBL, could be the first step toward improving treatment and prognosis. In accordance with infants with BCP-ALL, children between 0 and 1 years old with BCP-LBL have an inferior prognosis. Searching for *KMT2A* rearrangements in BCP-LBL infants should be mandatory in all patients.

## Figures and Tables

**Table 1:** Patient features of 210 BCP-LBL patients (0–18 years old) at diagnosis compared to 154 consecutively included Dutch BCP-ALL patients (1–18 years old).

Feature	BCP-LBL 210 (100%)	BCP-ALL 154 (100%)	*p*-Value
Sex			0.4440
Males	120 (57)	80 (52)	
Females	90 (43)	74 (48)	
Age in years (cat)			<0.0001
0–1	10 (5)	-	
1–7	95 (45)	109 (71)	0.0070
7–12	61 (29)	21 (14)	0.0180
12–18	38 (18)	23 (15)	0.8770
Unknown	6 (3)	0 (0)	
Murphy stage			
I	19 (9)	-	-
II	41 (19)	-	-
III	75 (36)	-	-
IV	69 (33)	-	-
Unknown	6 (3)	-	-

**Table 2:** Localizations of BCP-ALL and BCP-LBL patients. Bone marrow (BM) involvement is >5%. Central nervous system (CNS) includes CNS2 and CNS3. BCP-LBL patients without extramedullary involvement had isolated BM and isolated CNS disease.

	Bone Marrow	Peripheral Blood	Hepato-and/or Splenomegaly	Central Nervous System	Extramedullary
*B-ALL*	100%	95%	80%	51%	32%
*B-LBL*	26%	2%	2%	20%	99%

## Data Availability

The data presented in this study are available on request from the corresponding author. The data are not publicly available due to privacy.

## Supplementary Materials

The following supporting information can be downloaded at: https://www.mdpi.com/article/10.3390/cancers14163895/s1, Figure S1: Countries combined with used treatment protocols; Figure S2: Event-free and overall survival; Table S1: Collected variables from all BCP-LBL patients at diagnosis.
