# Supplementary material for: Pediatric Precursor B-Cell Lymphoblastic Malignancies: From Extramedullary to Medullary Involvement
Source: Cancers (Basel). 2022 Aug 12;14(16):3895. doi: 10.3390/cancers14163895 (PMC9405801; doi:10.3390/cancers14163895)
Supplement: Supplementary file 1 [file cancers-14-03895-s001.zip › cancers-1809748-supplementary.pdf]

Supplementary Table S1*:* **Collected variables from all BCP-LBL patients at diagnosis.**

**Variables at diagnosis**
Country
Study group
Patient ID
Gender
Date of initial diagnosis
Age at diagnosis
Localizations
Blasts in bone marrow (%)
Blasts in peripheral blood (%)
CNS status
LDH level/unit
Leukocyte count
Hemoglobin level
Thrombocyte count
Cytogenetic results
FISH results
Flowcytometry results
St Jude stage
B-symptoms
PET-scan performed
Other clinical diseases
Treatment protocol
Complete remission
Disease free survival (months)
Relapse
Date of relapse
Death
Cause of death
Biopsied location
Biopsy result

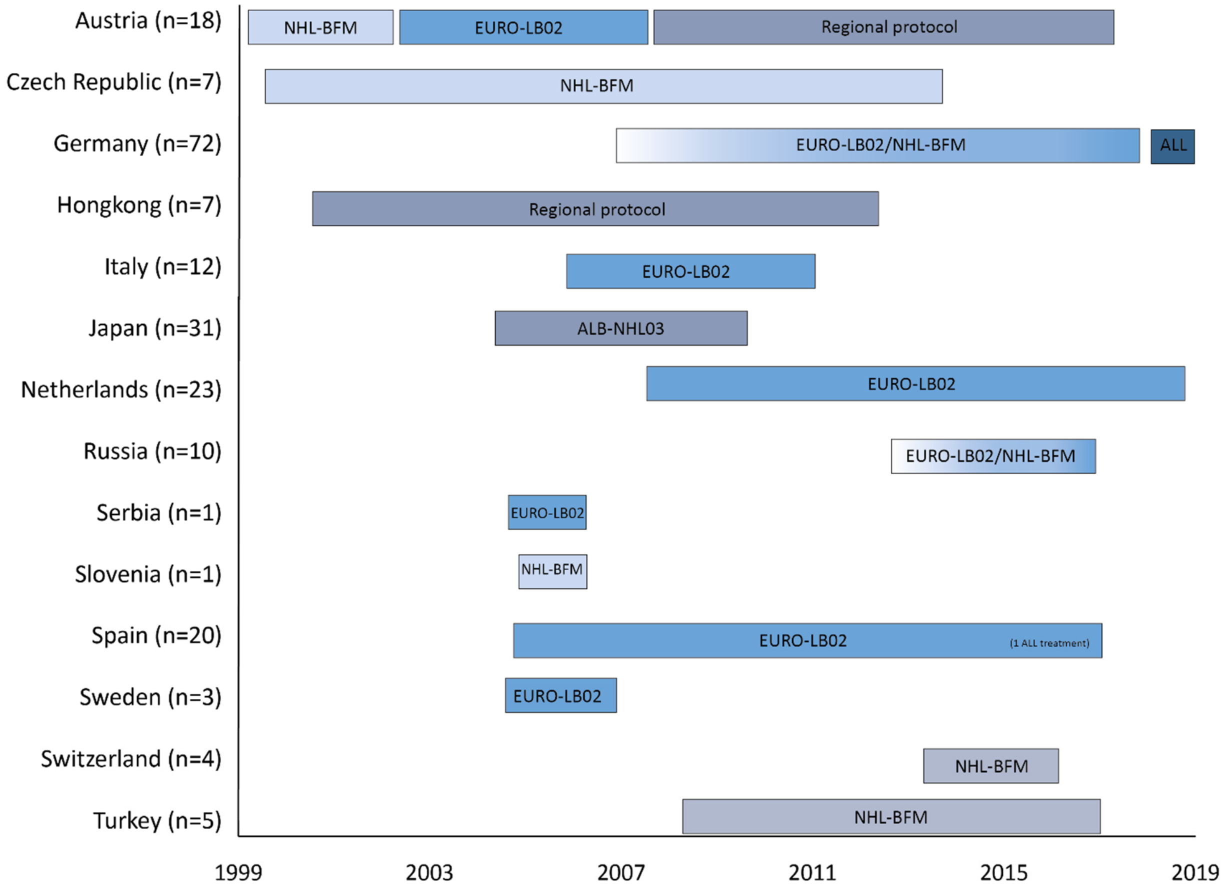

Supplementary Figure S1*:* **The 14 different countries combined with the used treatment protocols during these periods included NHL-BFM (n=67, dark grey), EURO-LB02 (n=80, light grey), ALB-NHL03 (n=31, white), ALL protocols (n=7) and regional protocols (n=15, white) that were similar as the EURO-LB02 protocol. Two ALL protocols were used.**

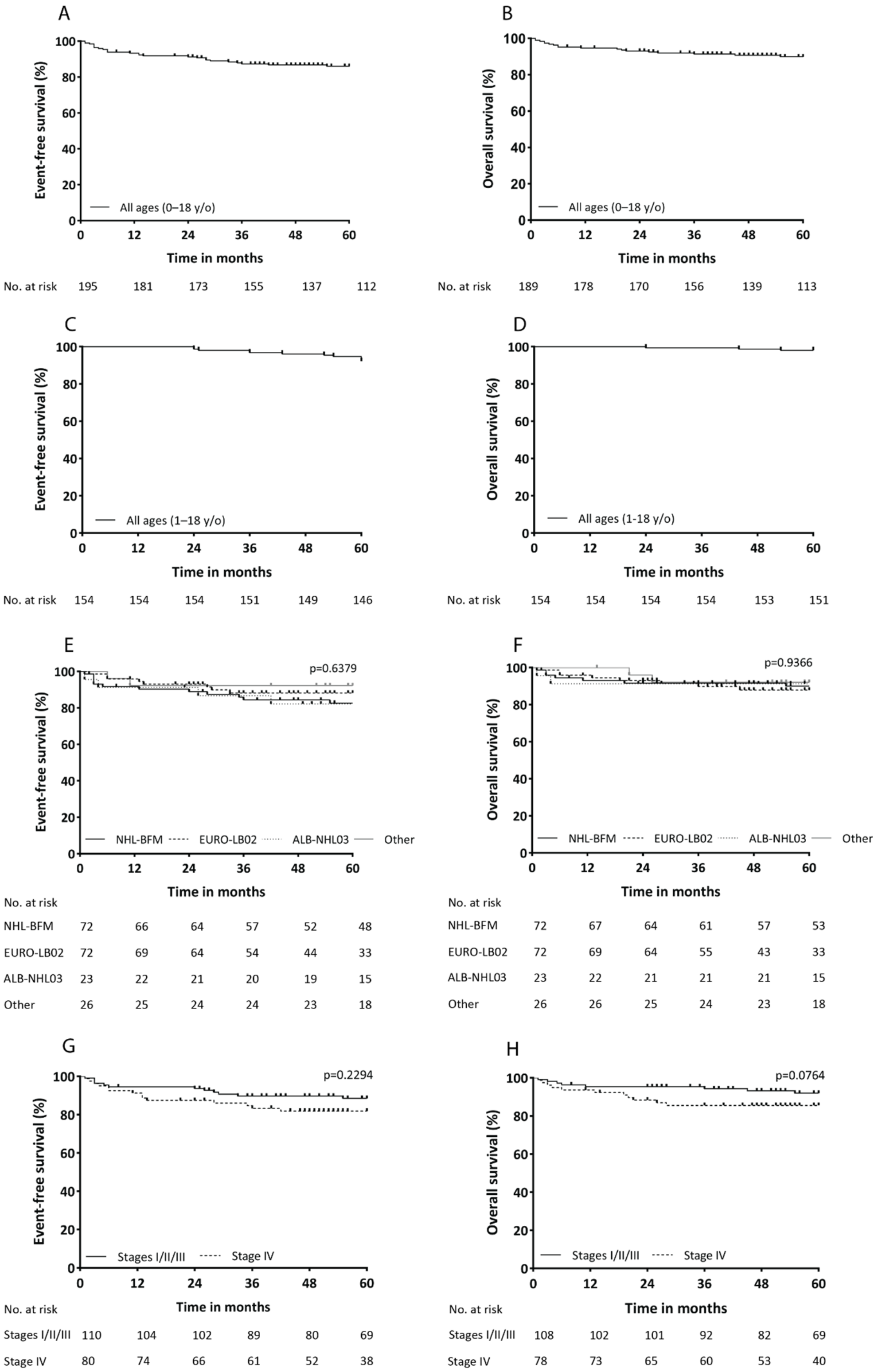

Supplementary Figure S2: **Event-free and overall survival for all BCP-LBL patients (A, B), all BCP-ALL patients (C, D), BCP-LBL patients treated according to different protocols (E, F), and BCP-LBL patients with different disease stages (G, H).**
